# Gelatin-layered and multi-sized porous β-tricalcium phosphate for tissue engineering scaffold

**DOI:** 10.1186/1556-276X-7-78

**Published:** 2012-01-17

**Authors:** Sung-Min Kim, Soon-Aei Yi, Seong-Ho Choi, Kwang-Mahn Kim, Yong-Keun Lee

**Affiliations:** 1 Department and Research Institute of Dental Biomaterials and Bioengineering, Yonsei University College of Dentistry, Seoul 120-752, South Korea; 2 Department of Periodontology, Yonsei University College of Dentistry, Seoul 120-752, South Korea

**Keywords:** β-tricalcium phosphate scaffold, multi-sized pores, gelatin coating, mechanical property, biological property

## Abstract

The multi-sized porous β-tricalcium phosphate scaffolds were fabricated by freeze drying followed by slurry coating using a multi-sized porous sponge as a template. Then, gelatin was dip coated on the multi-sized porous β-tricalcium phosphate scaffolds under vacuum. The mechanical and biological properties of the fabricated scaffolds were evaluated and compared to the uniformly sized porous scaffolds and scaffolds that were not coated by gelatin. The compressive strength was tested by a universal testing machine, and the cell viability and differentiation behavior were measured using a cell counting kit and alkaline phosphatase activity using the MC3T3-E1 cells. In comparison, the gelatin-coated multi-sized porous β-tricalcium phosphate scaffold showed enhanced compressive strength. After 14 days, the multi-sized pores were shown to affect cell differentiation, and gelatin coatings were shown to affect the cell viability and differentiation. The results of this study demonstrated that the multi-sized porous β-tricalcium phosphate scaffold coated by gelatin enhanced the mechanical and biological strengths.

## Introduction

Tissue engineering is one of the important methods of constructing biological tissues or devices for reconstruction and repair of the organ structures in order to maintain and improve their function [1]. The production of scaffolds, which are used for framework and initial support for the cells to attach, proliferate and differentiate, and form an extracellular matrix, is one area of tissue engineering [2]. The goal of scaffold production in tissue engineering is to fabricate reproducible, bioactive, and bioresorbable 3D scaffolds with appropriated properties that are able to maintain their structure for predictable times, even under load-bearing conditions [3]. The bioceramic scaffold is commonly used as a replacement of hard tissue through the 3D scaffold. The hydroxyapatite [HA], Ca_10_(PO_4_)_6_(OH)_2_, and β-tricalcium phosphate [β-TCP], Ca_3_(PO_4_)_2_, are well-known bioceramics which are biocompatible and bioactive. These materials exhibit a close resemblance in chemical composition to the human bone, a high biocompatibility with the surrounding living tissue, and high osteoconduction characteristics [4].

With the current tissue engineering, the scaffolds have suffered from limited cell depth viability when cultured *in vitro*, with viable cells existing within the outer 250 to 500 μm from the fluid-scaffold interface because of the lack of nutrient delivery into and waste removal from the inner regions of the scaffold construct [5,6]. To achieve better bioactive 3D scaffolds, bioceramic scaffolds with multi-pores were created to enhance biological properties as they have improved oxygen diffusion and fluid permeability.

The bioceramics have disadvantages of being brittle, and the composites of calcium phosphate ceramic with a protein-based polymer were of interest as the bone tissues repair materials due to their better mechanical properties as well as having adequate biological properties. The natural-based materials such as polysaccharides (starch, alginate, chitin/chitosan, hyaluronic acid derivatives) or proteins (soy, collagen, gelatin) in combination with a reinforcement of a variety of biofibers are one of the protein-based polymers, and the others are synthetic biodegradable polymers such as saturated poly(a-hydroxy esters), including poly(lactic acid), poly(-glycolic acid), and poly(lactic-coglycolide) copolymers [7]. Gelatin is obtained by thermal denaturation or physical and chemical degradation of collagen through the breaking of the triple-helix structure into random coils [8]. When compared with collagen, gelatin does not express antigenicity under physiological conditions; it is completely resorbable *in vivo*, and its physicochemical properties can be suitably modulated; furthermore, it is much cheaper and easier to obtain in concentrated solutions [9]. Gelatin is also clinically proven as a temporary defect filler and wound dressing because of its biodegradability and cytocompatibility [10,11]. However, the mechanical properties of gelatin itself are not satisfactory for hard tissue applications. Hence, the purpose of the present study was to create a 3D scaffold with enhanced mechanical and biological properties through multi-pore formation and gelatin coating.

## Materials and methods

### Preparation of multi-sized porous β-TCP scaffold and gelatin coating

The β-TCP scaffold was fabricated using template coating and freeze drying methods. The β-TCP slurry was made by dispersing the nano β-TCP powders (OssGen Co., Daegu, South Korea) into distilled water. The organic additives (5% polyvinyl alcohol, 1% methyl cellulose, 5% ammonium polyacrylate dispersant, and 5% *N*,*N*-dimethylformamide drying agent) were added to the slurry to improve the sintering force and to stabilize the scaffold structure. The polyurethane sponges used as template were coated with slurry and dried at room temperature or using the freeze drying method for 12 h, and the β-TCP scaffold was sintered at 1,250°C for 3 h. After the first coating, the micro-sized pore on the scaffold surface was fabricated by needle. The β-TCP scaffold was coated again with slurry and resintered. The final β-TCP scaffold size was 5 × 5 × 5 mm.

The 3% gelatin powder from the bovine skin was melted in distilled water at 45°C. After cross-linking with 0.2% glutaraldehyde, the gelatin was coated on the β-TCP scaffold through the dip-coating method at vacuum environment. Compressed air was blown into the β-TCP scaffold to remove the residual gelatin slurry. The gelatin-coated β-TCP scaffold was dried at 40°C in a vacuum drying oven for removal of the glutaraldehyde. The four types of sample were prepared by the above processes and designated with a code for the purpose of this paper [see Additional file 1].

### Characterization of the β-TCP scaffold

The surface morphologies of the sintered and gelatin-coated β-TCP scaffold were showed by a field emission scanning electron microscope [FE-SEM] (S-800; Hitachi, Tokyo, Japan) at an accelerating voltage of 20 kV. The detailed porosity and thickness of the structure were observed with micro-CT (Skyscan 1076; Skyscan Co., Antwerp, Belgium). The resolution was set at 9 μm, rotation step was 0.6° and rotation angle was 180°.

The compressive strength was measured by a universal testing machine (3366, Instron^® ^Co. Ltd. Norwood, MA, USA) at a crosshead speed of 1.0 mm/min. The compressive strength was calculated from the maximum load by the following equation:

S=F/A

where *S *is the compressive strength (in megapascals), *F *is the maximum compressive load (in newton), and *A *is the surface area of the β-TCP scaffold perpendicular to the load axis (in square millimeters).

### Biological evaluation

The biological properties were measured by cell proliferation and differentiation. The mouse osteoblast cell, MC3T3-E1 cell, (ATCC, Rockville, MD, U.S.A.) was used for *in vitro *tests. The cells (1 × 10^5 ^cells/100 μl) were seeded on each scaffold for 1, 3, 7, and 14 days in a 37°C, 5% CO_2 _incubator. The cell viability was measured by the Cell Counting Kit-8 [CCK-8] (Dojindo Laboratories, Kumamoto, Japan). The tetrazolium salt, 2-(2-methoxy-4-nitrophenyl)-3-(4-nitrophenyl)-5-(2,4-disulfophenyl)-2H-tetrazolium, monosodium salt (WST-8), was reduced by the dehydrogenases in the cells to show an orange-colored product (formazan). The absorbance was read at 450 nm with an ELISA reader (Benchmark Plus, Hercules, CA, USA).

The cell differentiation was measured by measuring the level of alkaline phosphatase [ALP] activity using the Sensolyte^® ^pNPP ALP Assay Kit (Anaspec, Inc., Fremont, CA, USA). The cells were lysed by Triton X-100 (Anaspec, Inc., Fremont, CA, USA) into the kit and reacted with the working solution. The final solution shows a yellow-colored product. The absorbance was measured at 405 nm.

## Results and discussion

### Characterization of the β-TCP scaffold

**Figure 1:**
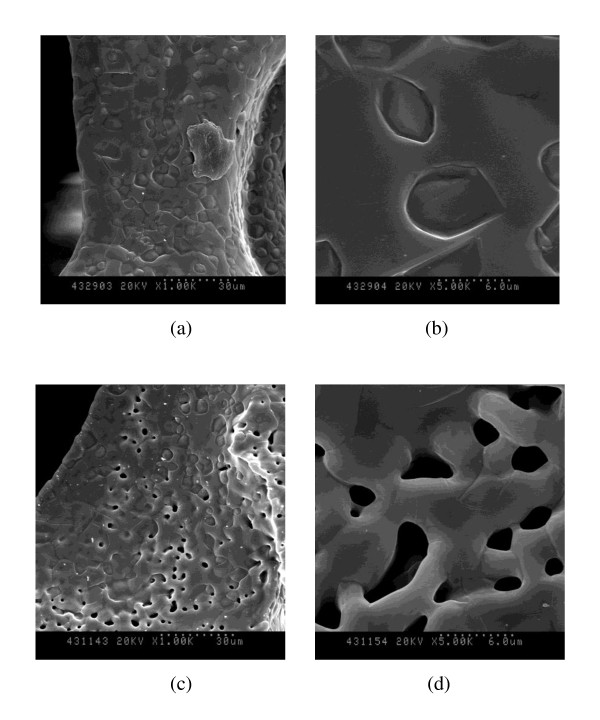
**SEM morphologies of the (a, b) SP and (c, d) MP surfaces**. Magnification of (a) and (c) is ×1,000 and of (b) and (d) is ×5,000.

**Figure 2:**
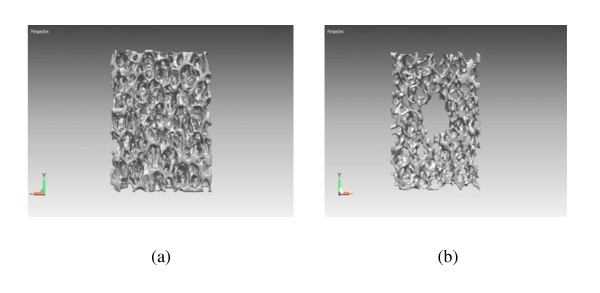
**Images of the cross-section of β-TCP scaffold with (a) SP and (b) MP**.

Figure 1 shows the surface morphologies of the β-TCP scaffolds that were not coated by gelatin. It was noticed that while the SP (Figures 1a,b) had a dense surface, the MP (Figures 1c,d) fabricated by freeze drying methods had micro-size pores on the surface. The micro-CT results have shown that the TCP had a similar pore size at all of the cross-section area (Figure 2a), whereas MP had a macro-size pore in the middle of the β-TCP scaffolds (Figure 2b). Table 1 in Additional file 1 shows the porosity and mean structure thickness of all samples. The porosities of SP, MP, SPGC, and MPGC were 78.04 ± 1.58, 82.65 ± 4.17, 77.29 ± 0.68, and 85.83 ± 1.02%, respectively. The mean structure thicknesses were 116.83 ± 6.18, 122.40 ± 12.39, 124.93 ± 4.29, and 112.90 ± 4.14 μm in SP, MP, SPGC, and MPGC, respectively. The porosity and mean structure thickness were similar between gelatin-coated and uncoated samples.

**Figure 3:**
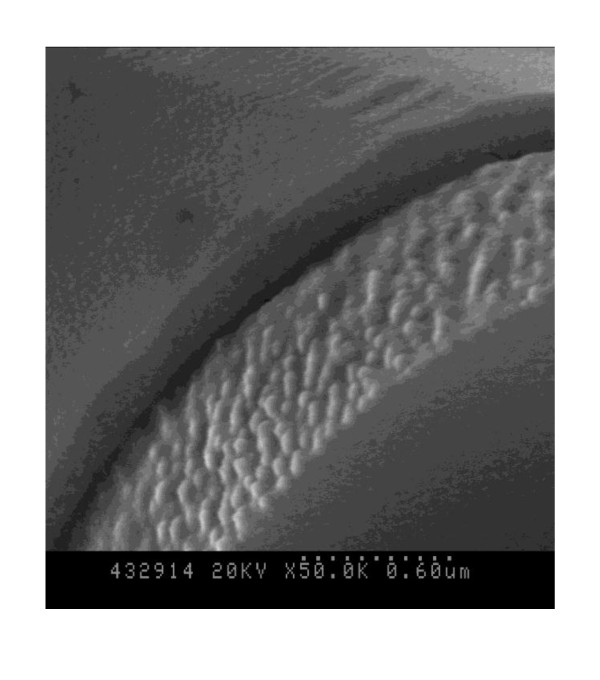
**SEM morphologies of the β-TCP scaffold surface coated by gelatin**.

**Figure 4:**
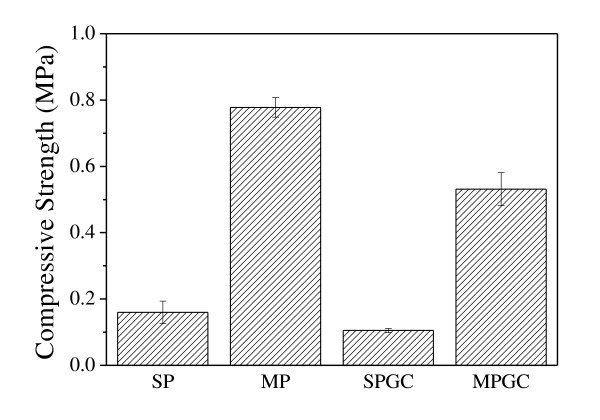
**The compressive strength of β-TCP scaffolds**.

Figure 3 shows the β-TCP scaffolds coated by gelatin. As shown by Figure 3, the gelatin was uniformly coated on the surface of the β-TCP scaffold with thickness around 180 nm. The compressive strength was measured by a universal testing machine and shown in Figure 4. The maximum compressive strengths were 0.15 ± 0.03, 0.11 ± 0.01, 0.78 ± 0.03, and 0.53 ± 0.05 MPa in SP, MP, SPGC, and MPGC, respectively. The compressive strength of the gelatin-coated scaffolds was about five times higher than that of the non-coated scaffolds. Most of the other studies using the mixed form of bioceramics and gelatin showed that the compressive strength was increased about two to four times [9,12,13]. The gelatin coating maintained the porosity and structure thickness of the scaffold which is similar to the uncoated scaffold. However, the high elasticity of gelatin as a polymer enhanced the compressive strength of the scaffold [14,15].

### Biological properties of the β-TCP scaffold

**Figure 5:**
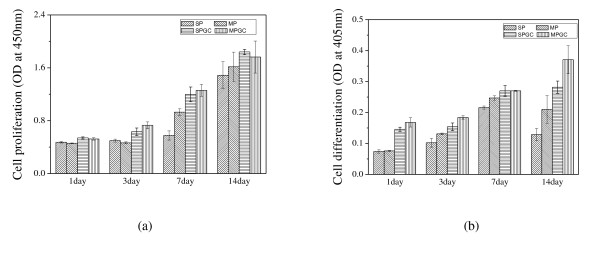
**Proliferation and differentiation of MCT3-E1 cells**. The (**a**) proliferation and (**b**) differentiation of MCT3-E1 cells on the β-TCP scaffolds after 1, 3, 7, and 14 days.

Figure 5a shows the cell viability results of the scaffolds following 1, 3, 7, and 14 days of culturing that was measured using the CCK-8 assay. The cells on the scaffolds continued to proliferate. The optical density value was similar between SP and MP and between SPGC and MPGC. This result shows that the multi-sized pores did not affect cell viability. However, the cell viability results on the gelatin-coated scaffolds were higher than those on the uncoated scaffolds. Hence, it was evident that gelatin coating enhanced the cell viability.

Figure 5b shows the ALP activity of the seeded cells on each scaffold. The MP and MPGC having multi-sized pores have shown a higher ALP activity compared to the scaffolds having uniformly sized pores. In addition, the gelatin coating on the scaffold enhanced the ALP activity compared to the uncoated samples. After 14 days, the MPGC showed the highest ALP activity than the others. This result, wherein the ALP activity was enhanced by increasing gelatin content, is in agreement with the previous research by Takahashi et al. [16].

## Conclusion

The scaffold having multi-sized pores were successfully fabricated using template coating and freeze drying methods. The gelatin-coated scaffold was fabricated uniformly by dip coating. The compressive strength of the β-TCP scaffold was enhanced about five times by gelatin coating. The scaffold having multi-sized pores resulted in improved cell differentiation, and gelatin coating enhanced the cell proliferation and differentiation. This study provides significant data regarding the mechanical and biological properties of the β-TCP scaffold according to the multi-sized pores and gelatin coating.

## Competing interests

The authors declare that they have no competing interests.

## Authors' contributions

SMK carried out the overall experiments including characterization of the scaffold as well as biological evaluation as the first author. SAY was in charge of cell culture. SHC participated in the biological evaluation and performed the statistical analysis. KMK participated in the biological evaluation. YKL conducted the design and analysis of all experiments as a corresponding author. All authors read and approved the final manuscript.

## Supplementary Material

Additional file 1 **Designation code, porosity, and mean thickness**. A table showing the designation code, porosity, and mean thickness of the structure of the samples. Click here for file
