# Supplementary material for: Gelatin-layered and multi-sized porous β-tricalcium phosphate for tissue engineering scaffold
Source: Nanoscale Res Lett. 2012 Jan 17;7(1):78. doi: 10.1186/1556-276X-7-78 (PMC3292831; doi:10.1186/1556-276X-7-78)
Supplement: Additional file 1 — Designation code, porosity, and mean thickness. A table showing the designation code, porosity, and mean thickness of the structure of the samples. [file 1556-276X-7-78-S1.DOC]

Table 1. The designated code, porosity and mean thickness of strut of the sample

| Designated Code | Description of the sample | Porosity (%) | Mean thickness of strut (µm) |
| --- | --- | --- | --- |
| SP | β-TCP scaffold with single pores /  without gelatin coating | 78.04±1.58 | 116.83±6.19 |
| MP | β-TCP scaffold with multi-sized pores /  without gelatin coating | 77.29±0.69 | 124.93±4.29 |
| SPGC | β-TCP scaffold with single pores /  with gelatin coating | 82.65±4.16 | 112.90±4.14 |
| MPGC | β-TCP scaffold with multi-sized pores /  with gelatin coating | 85.83±1.02 | 122.40±12.39 |
